# BslA-stabilized emulsion droplets with designed microstructure

**DOI:** 10.1098/rsfs.2016.0124

**Published:** 2017-06-16

**Authors:** Keith M. Bromley, Cait E. MacPhee

**Affiliations:** School of Physics and Astronomy, University of Edinburgh, James Clerk Maxwell Building, Peter Guthrie Tait Road, Edinburgh EH9 3FD, UK

**Keywords:** BslA, emulsions, microstructure, interfacial stabilization, arrested coalescence

## Abstract

Emulsions are a central component of many modern formulations in food, pharmaceuticals, agrichemicals and personal care products. The droplets in these formulations are limited to being spherical as a consequence of the interfacial tension between the dispersed phase and continuous phase. The ability to control emulsion droplet morphology and stabilize non-spherical droplets would enable the modification of emulsion properties such as stability, substrate binding, delivery rate and rheology. One way of controlling droplet microstructure is to apply an elastic film around the droplet to prevent it from relaxing into a sphere. We have previously shown that BslA, an interfacial protein produced by the bacterial genus *Bacillus*, forms an elastic film when exposed to an oil- or air–water interface. Here, we highlight BslA's ability to stabilize anisotropic emulsion droplets. First, we show that BslA is capable of arresting dynamic emulsification processes leading to emulsions with variable morphologies depending on the conditions and emulsification technique applied. We then show that frozen emulsion droplets can be manipulated to induce partial coalescence. The structure of the partially coalesced droplets is retained after melting, but only when there is sufficient free BslA in the continuous phase. That the fidelity of replication can be tuned by adjusting the amount of free BslA in solution suggests that freezing BslA-stabilized droplets disrupts the BslA film. Finally, we use BslA's ability to preserve emulsion droplet structural integrity throughout the melting process to design emulsion droplets with a chosen shape and size.

## 1. Introduction

Typically, liquid droplets adopt a spherical morphology in order to minimize their surface area and hence minimize their surface energy. However, this process can be arrested to give non-spherical droplet morphologies if an opposing elasticity is applied, either inside or at the surface of the droplet. For example, elasticity can be introduced internally by growing or encapsulating rigid filaments within emulsion droplets, as has been demonstrated in aqueous drops using actin filaments [1] and in oil droplets using wax crystals [2,3]. In the latter case, it was shown that anisotropic droplet morphologies could be arrested by the internal rigidity. Similarly, it is known that highly anisotropic morphologies result from arrested partial coalescence between solid fat droplets in ice cream [4]. *Interfacial* stabilization of anisotropic liquid droplets and air bubbles has been achieved using a variety of different methods. Jamming of colloidal particles at interfaces has been shown to stabilize a range of non-spherical arrested morphologies, including anisotropic emulsion droplets [5–7] and air bubbles [8], bicontinuous structures (bijels) [9] and recently water-in-air ‘liquid plasticine’ [10]. Anisotropic droplets have also been created using systems that do not use colloidal particles. For example, faceted droplets and droplets with tails have been formed via interfacial freezing of hexadecane and a surfactant; in this system, the interfacial elastic energy could overcome the near-zero interfacial tension [11,12]. Proteins have also been used to stabilize anisotropic structures. Specifically, a class of fungal proteins called hydrophobins were able to stabilize anisotropic air bubbles [13] and emulsion droplets [14], as well as being able to deform sessile drops of water in air [15].

BslA is a bacterial protein from *Bacillus subtilis* [16–19] that is similar in function to the fungal hydrophobins, although it is distinct in terms of structure, sequence and mechanism of action [20,21]. *In vitro* studies elucidated that the purified protein is surface active due to a conformational change that occurs only once the protein encounters a hydrophobic interface [21]. Further, we have previously shown that BslA can form an elastic film at both oil–water and air–water interfaces [20,21] and that the elastic film can stabilize anisotropic air bubbles [22]. Such qualities make BslA an excellent candidate for stabilizing emulsions. Indeed, the study that demonstrated the conformational change at the oil–water interface used BslA-stabilized emulsions for that purpose [21], and functionalized emulsion ‘microcapsules’ have been prepared using BslA as the stabilizer [23].

Here, we have created anisotropic liquid emulsion droplets with an elastic BslA surface. Initially, we demonstrate BslA's ability to arrest dynamic emulsification processes, such as droplet elongation, break-up and coalescence. Thus, by varying emulsification conditions, we can broadly control the resulting droplet morphology. Cooling the dispersed phase to solidify the droplets and then partially coalescing them achieved further control over emulsion microstructure. Such droplets could retain their partially coalesced structures during and after melting. Finally, to exert control over the final emulsion architecture, we created moulded fat droplets by casting them in a cylindrical template. The moulded fat droplets were released into a cold BslA solution and subsequently warmed to melt the internal fat phase. Despite melting the fat, the development of an interfacial elastic BslA film allowed the droplets to retain their original moulded morphology.

## 2. Experimental

### 2.1. BslA preparation

The BslA used throughout this research was a form of BslA in which cysteine amino acids were replaced with alanine, to prevent the potential formation of disulphide-bonded oligomers. To achieve this, the two cysteine residues at positions 178 and 180 were replaced by site-specific mutagenesis, creating a C178,180A mutant (N. Stanley-Wall 2015, unpublished data). The protein was expressed and purified as described in [21]. All BslA solutions were prepared in 25 mM pH 7 phosphate buffer that had been filtered through a 0.22 µm filter.

### 2.2. Emulsion preparation

Emulsification of hexadecane into BslA solution was performed using three traditional methods: high shear mixing; vortex mixing; and probe sonication. For high shear mixing, an Ultra-Turrax T10 rotor-stator with a gap width of 0.3 mm and rotor speed of 30 000 r.p.m. was used. This corresponds to a shear rate of 20 000 s^−1^. Samples were prepared at a range of BslA concentrations (0.05–0.5 mg ml^−1^) and oil volume fractions (*ϕ*_o_) (0.01–0.5) and emulsified for 15 s unless specified. Vortex mixing was performed for 60 s using an IKA Vortex Genius 3 operating at 2500 r.p.m. Probe sonication was performed using a Sonics Vibra-Cell VCX-500 probe sonicator equipped with a 3 mm tapered titanium microtip. Sonication was performed in 1 s pulses (amplitude = 20%) with 5 s pauses between each pulse for a total sonication time of 30 s. Emulsions created using vortex mixing or sonication were prepared at a BslA concentration of 0.2 mg ml^−1^ and at *ϕ*_o_ = 0.2.

Two alternative emulsification methods were also performed: a rollerbank method and a ‘floccing’ method. In the rollerbank method, a vial was completely filled with 50% 0.2 mg ml^−1^ BslA solution/50% hexadecane (v/v). This vial was then placed on a Stuart SRT9 roller mixer rotating at 36 rpm for 24 h. In the floccing method, a 200 µl pipette tip was filled with 200 µl hexadecane and then transferred to a pipettor set to dispense 100 µl. One hundred microlitres of the hexadecane was then dispensed from the tip and replaced with 100 µl 0.2 mg ml^−1^ BslA. The BslA solution was then dispensed and refilled from the tip by rapidly manually aspirating at least 10 times to produce an emulsion.

Partial coalescence of frozen emulsion droplets was achieved by centrifuging emulsions at 17 000*g* in a Thermo Scientific Heraeus Fresco 21 microcentrifuge. Centrifugation was performed for 5 min at 5°C. In samples prepared for confocal laser scanning microscopy, the hexadecane phase contained 100 µM Nile Red.

### 2.3. Emulsion droplet moulding

Cylindrical droplets with a defined diameter of approximately 210 µm were produced by using a 27 G needle as a template. The entire templating process was performed in a cold room at 5°C. First, a vial of coconut oil was cooled to 5°C until it was completely frozen. Then, a 27 G needle was pushed into the frozen coconut oil. When the needle was removed, a cylinder of frozen coconut oil remained inside. This cylinder was physically ejected (using a copper wire) directly into cold BslA solution (0.2 mg ml^−1^) that had been pipetted into the cavity of a microscope slide. Once several cylinders had been produced, the sample was covered with a 22 × 50 mm coverslip and imaged under a microscope. After imaging, the sample was warmed to 30°C and the droplets were imaged again.

### 2.4. Laser diffraction particle size analysis

Emulsion droplet sizing was performed using a Beckman–Coulter LS 13 320 particle size analyser. Emulsion samples were pipetted into 25 mM pH 7 phosphate buffer (stirred) in a cuvette until an obscuration value of approximately 10% was reached. Samples were measured at least three times with a measurement time of 60 s and the average size distribution was recorded. Particle size distributions were converted to the Sauter mean diameter, *d*_32_, also known as the volume–surface diameter as it represents the droplet size that reflects the volume : surface area ratio of the entire system.

### 2.5. Optical microscopy

Emulsion droplet morphologies were analysed using an Olympus BX-50 microscope equipped with a QImaging QICAM digital camera. Emulsions were diluted to *ϕ*_o_ = 0.001–0.005 depending on the size of the droplets and pipetted into the cavity of a microscope slide. A 22 × 50 mm coverslip was immediately placed over the top of the droplet to allow the droplets to cream onto the coverslip surface. In some experiments, the emulsion was pipetted directly into buffer solution in a cavity half-covered by a coverslip. This avoided elongation of large droplets at the air–water interface due to the application of the coverslip.

### 2.6. Confocal laser scanning microscopy

Hexadecane emulsion samples were prepared containing 100 µM Nile Red dye. An Instec TSA02i temperature stage was used to adjust the temperature during melting experiments. A 7 µl drop of emulsion at *ϕ*_o_ = 0.01 was placed on 22×50 mm coverslips with 45 µm thick tape used as a spacer. The samples were then covered with an 18×18 mm coverslip. In temperature-controlled experiments, slide assembly and sample mounting was performed *in situ*. A Zeiss LSM 700 confocal laser-scanning microscope was used to image the fluorescently labelled emulsion droplets. Samples were excited with a 10 mW, 555 nm solid-state laser attenuated to 10% intensity.

## 3. Results and discussion

Emulsions prepared using BslA as a stabilizer generally formed a mixture of spherical and aspherical droplets. The morphology of the aspherical droplets varied depending on the conditions and emulsification method used.

**Figure 1.**
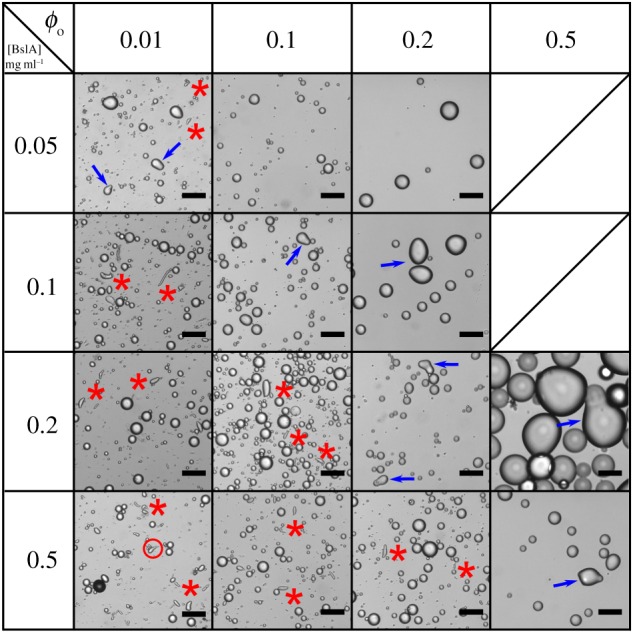
Emulsions prepared at different BslA concentration and oil volume fraction conditions by mixing in a high shear mixer for 15 s. The morphologies of the non-spherical droplets change as the system changes from the emulsifier-rich to the emulsifier-poor regime. Under emulsifier-rich conditions, rod-shaped droplets are stabilized (indicated by red asterisks). In the bottom-left panel, a teardrop structure is circled in red. Snowman droplets (indicated by blue arrows) are not observed under emulsifier-rich conditions, but appear under emulsifier-poor conditions. Rod-shaped droplets are not observed under emulsifier-poor conditions. The blank panels denote that a stable emulsion was not formed. Scale bars, 50 µm.

**Figure 2.**
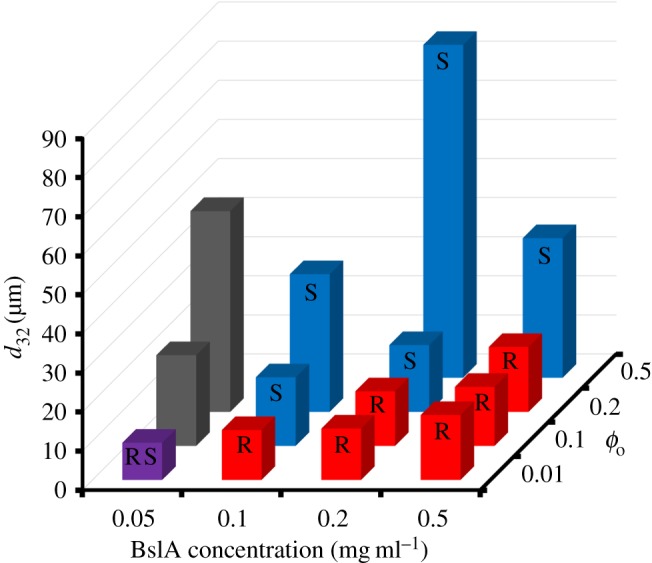
Laser diffraction particle size analysis of emulsions prepared at different BslA concentration and oil volume fraction conditions by mixing in a high shear mixer for 15 s. The colour of and the letters on the bars indicate the main anisotropic droplet morphology that was observed in each emulsion: rods (red, R), snowmen (blue, S), both morphologies (purple, RS) and neither morphology (grey).

Hexadecane emulsions were prepared by high-shear mixing at [BslA] = 0.05, 0.1, 0.2 and 0.5 mg ml^−1^ and *ϕ*_o_ = 0.01, 0.1, 0.2 and 0.5. Figure 1 shows representative images of the emulsions and figure 2 shows particle sizing data for each preparation condition. At the lowest oil volume fraction *ϕ*_o_ = 0.01, all four emulsion samples contained rod (red asterisks) and teardrop-shaped droplets, while partially coalesced ‘snowman’-shaped droplets were also seen at the lowest BslA concentration (0.05 mg ml^−1^; blue arrows). At this oil volume fraction, *d*_32_ increased slightly with increasing BslA concentration; however, we also observed the formation and stabilization of a foam when mixing high concentrations of BslA at low volume fractions of oil, thus BslA-stabilized air bubbles in the emulsion are likely to have skewed the data.

On increasing the hexadecane content to *ϕ*_o_ = 0.1, rod-shaped droplets were the dominant anisotropic droplet at [BslA] = 0.2 and 0.5 mg ml^−1^. At 0.1 mg ml^−1^ protein, rods were no longer observed, but ‘snowman’ morphologies were present. Only spherical droplets were observed at [BslA] = 0.05 mg ml^−1^ and these were larger (*d*_32_ > 20 µm) than the emulsions prepared at the same oil volume fraction but higher protein concentration.

At *ϕ*_o_ = 0.2, rod-shaped droplets were only observed at the highest BslA concentration. At [BslA] = 0.2 mg ml^−1^ snowman droplets could be seen. At [BslA] = 0.1 mg ml^−1^, most of the aspherical droplets appeared ovoid in shape. Only spherical droplets were observed at [BslA] = 0.05 mg ml^−1^. The *d*_32_ value decreased considerably with increasing BslA concentration from approximately 50 µm at 0.05 mg ml^−1^ to approximately 15 µm at 0.2 and 0.5 mg ml^−1^.

Only the two higher BslA concentrations could stabilize emulsions at *ϕ*_o_ = 0.5. Both emulsions contained snowman morphologies and no rods, while *d*_32_ was far greater than observed for those BslA concentrations at lower *ϕ*_o_.

From figure 1, it is clear that, alongside spherical emulsion particles, two types of anisotropic droplet are typically stabilized during emulsification by high shear mixing—rods or snowmen. Teardrops are also sometimes observed alongside rods (figure 1, bottom left panel). Rods are stabilized when the BslA : oil ratio is high enough. Under conditions where less BslA is available for adsorption, either due to a lower BslA concentration or increased *ϕ*_o_, rods are no longer observed and snowman structures are instead seen. These observations are consistent with predicted emulsification mechanisms under ‘emulsifier-rich’ and ‘emulsifier-poor’ conditions [24].

In the emulsifier-rich regime, there is sufficient emulsifier to stabilize droplets during droplet break-up such that the smallest droplets achievable under the chosen hydrodynamic conditions are fully coated in protein. When a droplet splits in two, a small filament develops as the droplet is stretched. The filament ultimately breaks apart and the resultant droplets that form from the pinched-off filament are usually identified as ‘satellite droplets’ [25,26]. In the presence of sufficient BslA, these filaments are stabilized. The presence of other anisotropic drop shapes such as ‘teardrops’ and larger elongated drops (figure 1, bottom left panel) supports the idea that the rods are indeed a product of the droplet break-up process as remnants of the ‘mother drop’ would necessarily be formed with the satellite drops. Importantly, as the products of droplet break-up quickly develop a full monolayer of BslA, partial coalescence of colliding droplets is inhibited and thus snowman structures are not usually observed.

In the emulsifier-poor regime, there is no longer sufficient BslA present to stabilize the products of droplet break-up, so rods and teardrops are no longer observed under high shear mixing. High shear mixing creates high and low perturbation zones during the emulsification process [27]. In the high shear zone, the shear rate is 20 000 s^−1^, which corresponds to a timescale of droplet deformation and break-up of roughly 50 µs [28]. This short droplet deformation and break-up time decreases the probability that BslA could stabilize the transient structures formed via droplet break-up under emulsifier-poor conditions. However, in such emulsifier-limiting conditions, many of the droplets would be partially coated [24] as they emerge from the high shear zone. When two droplets *partially* coated in BslA collide, the coalescence is quickly arrested by the BslA layer and a snowman droplet is created. The Smoluchowski expression for collision rate in laminar flow [29] estimates that each droplet would experience roughly 5000 collisions s^−1^ (for droplet diameter = 16 µm, *ϕ*_o_ = 0.2, shear rate, 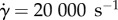) within the high shear zone. Given the dimensions of the rotor-stator head, the high shear zone in our system accounts for roughly 5% of the total emulsion volume. If the majority of collisions resulted in partial coalescence, we would expect to observe a much higher yield of snowmen. The low yield of snowmen droplets suggests that partial coalescence can only occur over a limited range of surface fractions of colliding droplets. Indeed, this surface fraction range has been previously estimated to be between approximately 0.7 and 0.9 for droplets colliding in a particle-stabilized emulsion system [5]. Below an average surface fraction of 0.7, coalescence could not be arrested and above a surface fraction of 0.9 on an individual droplet, coalescence would not occur. Given the low yield of snowmen, it seems likely that colliding BslA-stabilized droplets have similar limits to the arrest of coalescence as particle stabilized emulsions [2,5,30]. At extremely low BslA concentrations, the partially coated droplets have so little BslA adsorbed at the surface that the snowman morphology can no longer be arrested. This lower limit of anisotropic shape stabilization occurs at roughly 0.05 mg ml^−1^ for *ϕ*_o_ = 0.1–0.2 (figure 1).

**Figure 3.**
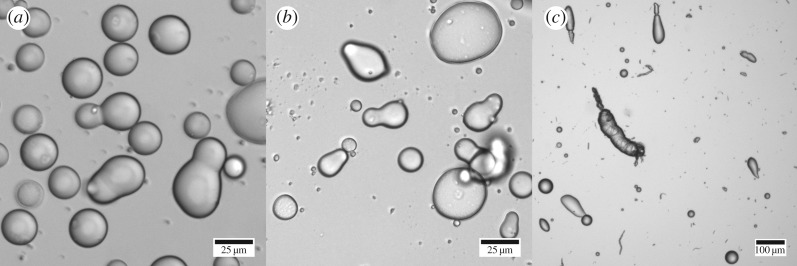
(*a*) Hexadecane snowman droplets formed via emulsification using a probe sonicator. (*b*) Hexadecane snowman droplets formed via emulsification using a high-shear mixer at a shear rate of 20 000 s^−1^. (*c*) Hexadecane teardrops and rods formed via emulsification using a high shear mixer at a shear rate of 5000 s^−1^. All three emulsions (*a–c*) were prepared at [BslA] = 0.2 mg ml^−1^ and *ϕ*_o_ = 0.2.

When emulsions were prepared via sonication, the predominant aspherical droplets formed also had the snowman morphology (figure 3*a*). There are two major parallels between the high shear mixing technique and emulsification by probe tip sonication. First, a high-intensity cavitation zone develops around the probe tip, leaving a ‘dead zone’ closer to the edge of the sonication vessel [31], much like the high and low shear zones that exist in a high shear mixer. Second, the timescale of cavitation bubble collapse is of the order of less than 1 µs [32], which is even shorter than the timescale of droplet deformation and break-up during high shear mixing (50 µs for a shear rate of 20 000 s^−1^ [28]). As such, under the same emulsifier-poor conditions, it is not surprising that droplet break-up cannot be stabilized during sonication and thus a similar droplet morphology is produced from both sonication and high-shear mixing (figure 3*a,b*).

The emulsifier-poor conditions that produced snowman droplets in the high shear mixer ([BslA] = 0.2 mg ml^−1^ and *ϕ*_o_ = 0.2) could not stabilize rods as the droplet break-up process occurred too rapidly for the low concentration of available protein to stabilize. If the droplet break-up process was slowed by changing the emulsification method, could rods be stabilized under the same emulsifier-poor conditions?

By lowering the shear rate from 20 000 s^−1^ to 5000 s^−1^, emulsions prepared at [BslA] = 0.2 mg ml^−1^ and *ϕ*_o_ = 0.2 had rod and teardrop morphologies (figure 3*c*) instead of the snowman morphology that was prevalent at the higher shear rate.

**Figure 4.**
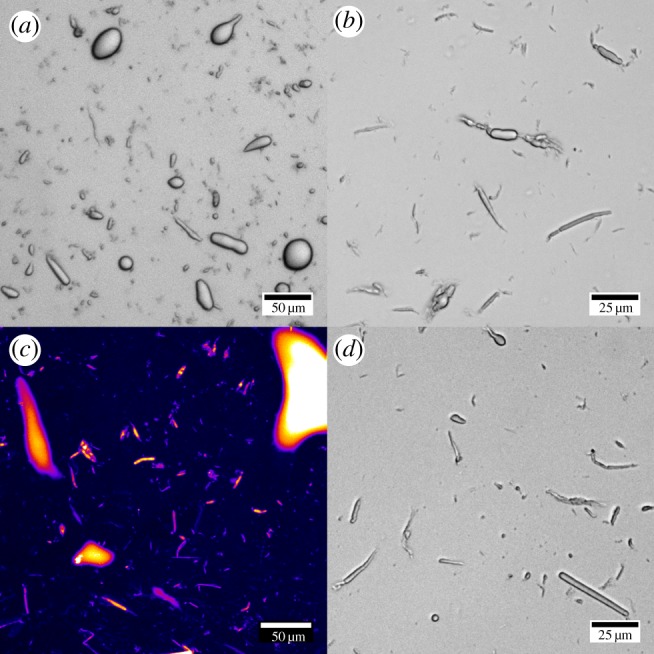
Images of emulsion droplets arrested during emulsification. (*a*) Elongated droplets trapped during vortex mixing at [BslA] = 0.2 mg ml^−1^ and *ϕ*_o_ = 0.2. Note the presence of a teardrop-shaped droplet near the top of the image. (*b*) Rod-shaped droplets formed by rolling a vial of BslA and hexadecane on a rollerbank for 24 h. (*c*) Rod-shaped rollerbank droplets stained with Nile Red and viewed using confocal laser-scanning microscopy. (*d*) Rod-shaped oil droplets created by ‘floccing’ BslA solution in a pipettor tip in the presence of hexadecane.

Equally, preparing emulsions by shaking on a vortex mixer created long rod-shaped emulsion droplets with aspect ratios as high as 50 (figure 4*a*). The widths of the rod-shaped droplet in these samples ranged from approximately 1 to 10 µm. As with rods formed under high shear mixing, teardrop-shaped emulsions were observed alongside the rod-shaped droplets (figure 4*a*). High aspect ratio droplets were also formed by slowly rolling a vial containing oil and BslA solution on a rollerbank for 24 h. In that case, only a small percentage of the oil was emulsified, but the droplets that formed were extremely small, with the lateral diameter of the rods often measuring below 1 µm (figure 4*b*). Confocal laser-scanning microscopy of the droplets stained with Nile Red established that they were indeed oil droplets (figure 4*c*) and not BslA stabilized rod-shaped air bubbles as have been observed previously [22]. Repeatedly shearing BslA in the presence of hexadecane in a pipettor tip (the floccing method, see Experimental) produced rod-shaped droplets of a similar size and aspect ratio (figure 4*d*) to the droplets created by the rollerbank method.

The stabilization of rods by vortex mixing under emulsifier-poor conditions ([BslA] = 0.2 mg ml^−1^, *ϕ*_o_ = 0.2) (figure 4*a*) can be explained by considering the shear profile in the system. Vortex mixers have a relatively large shear zone that leads to long residence times [27] and a lower shear rate compared with high shear mixers. Those differences result in a slower droplet break-up process as the timescale of droplet deformation is inversely proportional to the shear rate [28]. We suggest that in this case the droplet break-up process is slowed enough for BslA to be able to stabilize the filament and teardrops that develop as a droplet breaks apart even when in the emulsifier-poor regime.

Rods are also the predominant anisotropic droplet formed via the rollerbank method (figure 4*b*,*c*). Typically, the production of extremely fine emulsion droplet sizes with diameters of the order of a few micrometres requires the entire bulk oil phase (for oil-in-water emulsions) to be broken up and suspended in the aqueous phase, a high-energy process. The rollerbank process is, in contrast, a low-energy method that produced fine oil droplets directly from the bulk phase without breaking down the bulk oil phase entirely. We hypothesize that the ability to generate emulsion droplets by gently rotating separate hexadecane and aqueous BslA phases at a 1 : 1 ratio on a rollerbank is the result of variations in shear stress within the BslA nanofilm formed at the oil–water interface. In regions of increasing shear stress such as when the BslA nanofilm approaches the vial wall, the BslA nanofilm would fracture as the shear stress applied to the interface would result in extensile stress *within* the interface. Fracturing would expose fresh oil–water interface for excess BslA in the aqueous phase to adsorb onto, thus increasing the total surface area of interfacially bound BslA. The increase in interfacial surface area would ultimately lead to the formation of wrinkles at the interface, which we suggest close in on themselves and bleb off the interface as rod-shaped droplets. This mechanistic idea was supported by the observation that the floccing method, a different way of applying shear stress, also produced small rod-shaped droplets with very similar size and morphology to the droplets generated by the rollerbank method (figure 4*d*). In this case, a BslA nanofilm could form along the inside of the pipettor tip, which would be wet by hexadecane. As we hypothesized for the rollerbank method, the BslA nanofilm at the oil–water interface within the pipette tip would experience similar extensile and compressive stresses during floccing, except in this case, the stress applied can be controlled by the rate of aspiration. Similar observations have been made by shearing BslA at an air–water interface, leading to the formation of a wide range of higher order BslA assemblies [19,22].

**Figure 5.**
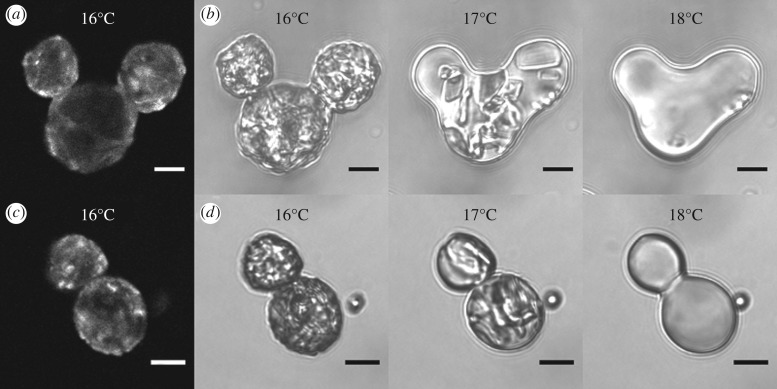
Melting transitions of BslA stabilized partially coalesced frozen hexadecane droplets with (*a*,*b*) no excess BslA and (*c*,*d*) 0.9 mg ml^−1^ BslA in the continuous phase; (*a*) and (*c*) are confocal images of the frozen droplets stained with Nile Red. The image sequences (*b*) and (*d*) are transmission images taken before melting at approximately 16°C, during melting at approximately 17°C, and after melting at approximately 18°C. Relaxation at the neck occurred more significantly in the absence of free BslA. The ramp rate was 0.5°C min^−1^. Scale bars, 10 µm.

Although they were the predominant type of non-spherical droplet observed in emulsifier-poor conditions, the overall yield of snowman structures formed by sonication and high-shear mixing was very low. By centrifuging BslA-stabilized hexadecane droplets, we could increase the incidence of droplet–droplet collisions, thus promoting the creation of snowman droplets. This was true whether or not we froze the hexadecane emulsion prior to centrifugation. Using emulsions that were frozen and then centrifuged, we observed the melting process to determine how efficiently BslA could maintain the shape of the partially coalesced droplets. Figure 5*a* shows a triplet of hexadecane droplets melting in a cleaned system where excess BslA had been removed. Although the overall ‘Mickey Mouse’ morphology of the triplet was maintained, significant relaxation at the joints could be observed. Relaxation at the joints was a common feature of melting doublets and triplets in systems with no excess BslA. This meant the final surface area of the melted doublets and triplets was significantly lower than it would be for two spherical oil droplets (with an equivalent total volume to the doublet or triplet) conjoined by a small neck. The relaxation of the neck suggests that the freeze–thaw process disrupts the interfacial BslA nanofilm. We propose that this is a consequence of the formation of planar crystalline surfaces at the frozen hexadecane droplet interface, resulting in a higher relative surface area per droplet. With no excess BslA available to adsorb onto the newly exposed hexadecane interface, the broken BslA nanofilm requires time of the order of a few seconds [21] to reorganize during the melting process. The overall decrease in droplet surface area implies that some BslA is lost from the interface during a freeze–thaw event. To test the idea that neck relaxation was a consequence of BslA reorganization and potentially loss of BslA during the freeze–thaw process, the experiment was repeated with excess BslA in the aqueous phase. As expected, far less relaxation was observed at the necks (figure 5*b*) demonstrating that although some rearrangement and/or loss of interfacially bound BslA still occurred, the free BslA in the aqueous phase could adsorb onto the bare hexadecane surfaces to repair the ruptured nanofilm. Ultimately, the availability of free BslA in the aqueous phase allowed for a more faithful replication of the original frozen morphology.

**Figure 6.**
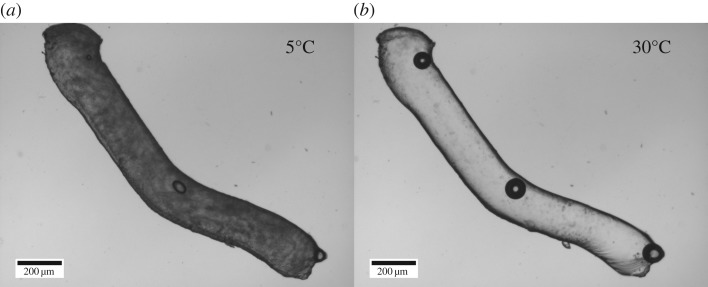
(*a*) Coconut fat rod-shaped emulsion drop formed by extruding the fat from a 27 G needle into BslA solution at 5°C. (*b*) The same droplet after warming to 30°C. Even after the fat had melted to oil, the overall morphology of the droplet was maintained by the elastic BslA film at the interface.

In the previous method, we demonstrated that BslA could preserve anisotropic droplet shapes while the internal oil phase was melted. However, the anisotropic snowman droplets were based on the coalescence of two or three preformed spherical droplets. Can this method of interfacial stabilization allow us to design a method that can produce droplets with specific morphologies and dimensions? Instead of creating an emulsion using one of the traditional methods, we used a 27 G needle with an inner diameter of approximately 210 µm to template a core of fat (cooled coconut oil, *T*_m_ ≈ 25°C) that was extruded directly into a cold BslA solution (*T* < *T*_m_ of the oil phase), thus negating any driving force towards the formation of spherical droplets. Upon melting the fat by warming to 30°C, the morphology of the coconut oil cylinders remained virtually identical to that of the original fat cylinders (figure 6) demonstrating that BslA had successfully formed an elastic interface around the large rod-shaped oil droplet. As observed with the melting snowman morphologies in the presence of excess BslA, a very small amount of relaxation occurred.

We have demonstrated that the interfacial protein BslA can be used to stabilize emulsions with programmed size and shape. This work could be developed to produce highly monodisperse anisotropic emulsions using microfluidics in an extension of the work of Xu *et al*. [33] which has shown that monodisperse cylindrical and discoid solid particles can be created by thermal setting within a microfluidic device. A similar microfluidic method has recently been exploited to create monodisperse anisotropic droplets stabilized by an internal crystal endoskeleton [34]. Using a 5 nm thick BslA layer to stabilize these emulsions offers several advantages over alternative systems. First, the emulsions do not require a solid scaffold to retain their anisotropic morphology, as is the case if anisotropic emulsions are stabilized using internal fat crystals [2,3], allowing the preparation of entirely liquid anisotropic emulsions with only a very thin protein sheet as a stabilizer. Second, the solid templating technique that we used to generate designed emulsion droplets is only possible if the film-forming stabilizer can either adsorb onto solid surfaces or adsorb onto a melting interface quickly enough to arrest droplet relaxation. The technique enables the creation of liquid emulsion droplets that are extremely close replicas of the originally cast solid shape. This is an improvement over a previous method of extruding liquid oil through capillaries that created elongated droplets with widths broadly similar to the capillary width [7], which relied on particles adsorbing to a liquid–liquid interface. Third, BslA has a rich surface chemistry that should enable facile modification of the surface of the emulsions and proteins offer the advantage of being genetically modifiable to allow specific functionalization of the emulsion surface. This has already been demonstrated to create fluorescently labelled BslA-stabilized emulsions [23].

This control over droplet morphology should lead to the development of methods to produce emulsions with defined microstructure in the future. However, production of commercial formulations generally requires high throughput methods and low-throughput templating methods may be unachievable in an industrial setting, so developing an understanding of how BslA influences droplet structure during dynamic emulsification processes is also important. Here, we have demonstrated how and why different emulsification methods produce different emulsion morphologies. With this knowledge, it should be possible to design protocols to produce emulsion populations with uniform microstructure. For example, by applying low shear in a flow-through system containing BslA and an oil, it should be possible to create almost exclusively rod-shaped emulsions in large batches. With such control over droplet morphology attainable through a variety of processes, incorporating BslA into functional emulsion formulations looks like a realistic prospect.
